# They need to be recognized as a person in everyday life: Teachers’ and helpers’ experiences of teacher–student relationships in upper secondary school

**DOI:** 10.3402/qhw.v11.31634

**Published:** 2016-10-04

**Authors:** Vibeke Krane, Bengt Karlsson, Ottar Ness, Per-Einar Binder

**Affiliations:** 1 Center for Mental Health and Substance Abuse, Faculty of Health Sciences, University College of Southeast Norway, Drammen, Norway; 2 Department of Clinical Psychology, University of Bergen, Bergen, Norway

**Keywords:** Teacher–student relationship, dropout, mental health, upper secondary school

## Abstract

The aim of this study was to explore how teachers and helpers experience that teacher–student relationship (TSR) is developed and promoted in upper secondary school.

We also explored their experiences of qualities of TSR with students with mental health problems or at risk of dropping out. The study used a qualitative and participative approach; key stakeholders were included as co-researchers. Focus group interviews were held with 27 teachers and helpers. A thematic analysis was conducted. The participants’ descriptions of important experiential dimensions of TSR were clustered around four themes: (1) to be recognized as a person with strengths and challenges in everyday life, (2) collaborative relationships between students and teachers, (3) flexible boundaries in the relationship between teachers and students and (4) organization of classes and procedures set the stage for TSR. Collaborative, emotional and contextual qualities were found important to the development of TSR in upper secondary school. Experiences of negative qualities of TSR can contribute to push students out of school. Teachers and helpers experience that TSR may have the potential to play a role in promoting mental health in students’ everyday life.

This article will explore teachers’ and helpers’ experiences of how teacher–student relationship (TSR) is promoted and developed in upper secondary school. We will also address TSR with students with mental health problems or at risk of dropping out. Schooling is central for young people, and the relationship between teachers and students is pivotal in students’ everyday lives. Thus, TSR has been a focus of both educators’ and researchers’ attention for decades (Pianta, Hamre, & Stuhlman, 2003; Sabol & Pianta, 2012).

The TSR develops through interaction and communication between teachers and students. Both attachment theory and developmental systems theory can be used to understand the concept of TSR. Children and youths’ different attachment styles are associated with their relationships with teachers (Pianta & Allen, 2008; Sabol & Pianta, 2012). In a developmental systems theory perspective, different multilevel systems (individual, family, classroom, peers, school organization and environment) interact in the development of TSR (Bronfenbrenner & Morris, 1998; Sabol & Pianta, 2012). Positive TSR is characterized by closeness, warmth and perceived support from teacher. Negative TSR is characterized by conflict, negative emotions and lack of report (Birch & Ladd, 1998; Drugli, 2013; Sabol & Pianta, 2012). There is substantial evidence that a positive TSR is crucial for students’ motivation, achievement and learning (Bergeron, Chouinard, & Janosz, 2011; Hattie, 2009; Nordenbo, Larsen, Tiftikçi, Wendt, & Østergaard, 2008; Roorda, Koomen, Spilt, & Oort, 2011). In addition, Roorda et al. (2011) argue that TSR is even more important for students’ academic adjustment, as they grow older. However, the quality of TSR is declining and less positive among older students and their teachers (Hamre & Pianta, 2001; Murray & Murray, 2004). School environment, education and TSR are important factors contributing to the developmental processes and the mental health of adolescents (Bronfenbrenner & Morris, 1998). Positive TSR has been associated with positive outcomes for students such as a reduction in depression and an improvement in self-esteem suggesting a potential of TSR as a promoting factor for youths’ mental health (Colarossi & Eccles, 2003; Cornelius-White, 2007; McGrath, 2009; Pianta et al., 2003; Wang, Brinkworth, & Eccles, 2013). In contrast, other studies have found that a negative TSR may act as a risk factor for student mental health by decreasing self-esteem and increasing depression (De Wit, Karioja, Rye, & Shain, 2011; Dods, 2013). Several studies have also found associations between students’ mental health problems and dropout from upper secondary school (De Ridder et al., 2013; Garvik, Idsoe, & Bru, 2014; Vander Stoep, Weiss, & Kuo, 2003). Dropout from upper secondary school is defined as a national problem and a political concern in several western countries (Lamb, Markussen, Teese, Sandberg, & Polesel, 2011). Young people without upper secondary education have fewer opportunities in the labor market and prospects of poorer physical and mental health (Croninger & Lee, 2001; De Ridder et al., 2013). Numerous studies have shown an association between TSR and dropout from upper secondary school by either preventing/decreasing dropout (Barile et al., 2012; Cornelius-White, 2007; Croninger & Lee, 2001; Lee & Burkam, 2003; Lessard, Butler-Kisber, Fortin, & Marcotte, 2014) or lowering the risk or intention for dropout (Bergeron et al., 2011; Frostad, Pijl, & Mjaavatn, 2015; McGrath, 2009; Muller, 2001).

Although several studies have found TSR important to both students’ mental health and dropout, there is a lack of knowledge on how TSR is experienced and developed in practice. Such knowledge is essential to develop and promote positive and healthy relationships between teachers and students. To gain this knowledge, we need a deeper understanding of different perspectives on the importance and awareness of TSR in upper secondary school. This involves exploring various qualities and aspects of TSR and how positive TSR can be developed with students with mental health problems or at risk of dropping out. Teachers in upper secondary school experience TSR on a daily basis, and their perspectives and subjective experiences on the importance of TSR and how it is developed are therefore important to explore. Bringing in other perspectives from professional helpers who work with these students like social workers, school nurses and school advisors will also give valuable contributions to knowledge development. The aim of this study was to explore how teachers and helpers experience that TSR is developed and promoted in upper secondary school. We also aimed to explore their experiences of qualities of TSRs particularly in relation to students with mental health problems or at risk of dropping out. To explore these issues, we raised the following research questions:

1. How do teachers and helpers experience that TSRs are developed and promoted in upper secondary school?
2. What do teachers and helpers experience as important relational qualities concerning students’ mental health and dropout in upper secondary school?

## Methodology

The study had a qualitative, descriptive and explorative design. The data were collected in focus groups, respectively, two groups with teachers and two groups with helpers (Kitzinger, 1994; Malterud, 2012). Thematic analysis was used to obtain and systemize the data (Braun & Clarke, 2006). In this study, we wanted to explore the participants’ subjective lived experiences of TSR. These experiences were generated and shared through intersubjective discourses and created through dialogs as words and stories in the interview setting (Borg, Karlsson, Lofthus, & Davidson, 2011). Thus, we have emphasized both an exploration of lived experience and a reflexive stance towards our own pre-understanding and the interview setting (Alvesson, 2003; Binder, Holgersen, & Moltu, 2012).

In a participatory approach, people with lived experiences are involved in the research process; this is described as a way of doing research *with* people instead of only *on* or *about* people (Borg & Kristiansen, 2009). The aim is to embrace multiple understandings of the studied phenomenon and to contribute to knowledge development through a collaborative process between people with lived experiences and researchers (Borg, Karlsson, Kim, & McCormack, 2012).The participatory approach in this study involved a young woman with lived experiences of dropout from upper secondary school who worked as a co-researcher in the study. She participated in the development of the study, in all interviews and in the data analysis. In line with the participatory approach, a competence group of key stakeholders contributed throughout the research process (Borg et al., 2011). The group consisted of two students, two teachers, two parents, a school nurse and a school psychologist. This competence group was involved in developing the research project, working on the interview guide, data analysis and discussions of how to conduct the study.

### Participants

The participants in this study were recruited by the management in two upper secondary schools and head of a psychosocial team for youth in the eastern part of Norway. A total of 27 people participated in the study: 15 teachers and 12 helpers. The inclusion criterion was teachers and helpers who had experience from work with students who had dropped out or were at risk of dropping out from upper secondary school. Ten of the participating teachers worked in specializing in general studies (SGS), two worked in vocational programs (VP) and three worked in both study paths. The teachers’ ages ranged from 26 to 64 years old, mean age was 46. Their work experience as teachers ranged from 0.5 to 35 years, mean 11.5 years. The participating helpers were school nurses, social workers, school psychologist, counselors and other school employees. All helpers had experience with working with students in both SGS and VP. The helpers’ ages ranged from 19 to 66 years old, mean age 45.5 years old. Their work experience ranged from 2 to 27 years, mean 9 years. As the recruiting county has a wide spread in demographic and social living conditions, the participants were recruited from different parts of the county to secure variety in the selection.

### Data collection

The data collection was conducted in the spring of 2014. Before the interview, the participants were informed that we were seeking to explore their subjective experiences with TSR related to students’ mental health and dropout. The participants were interviewed in four focus groups: two groups with teachers and two groups with helpers (Kitzinger, 1994; Malterud, 2012). As we wanted to facilitate an open dialog with exploration of the participant's experiences, we used semi-structured exploratory interviews (Binder, Moltu, Hummelsund, Sagen, & Holgersen, 2011). The first author conducted the interviews, whereas the co-researcher added questions and made notes. The interviews were audiotaped and transcribed verbatim. In one of the interviews, we had a problem with the recording, so parts of the interview were transcribed verbatim and the last part was written as an abstract in collaboration between the first author and the co-researcher.

### Thematic analysis

Thematic analysis was used to analyze the material according to the research questions (Braun & Clarke, 2006). The material was imported to the software program NVivo for organization and analysis. In the first step, the first author listened to the audiotapes and read all the interview transcripts to get an initial impression of the material. She noted down initial thoughts and reflections. In the next step, the meaningful units of the material such as illustrative quotations and descriptions were identified and coded into initial codes (Table I). For example, the meaningful unit: “To create a safeness … safeness through a relationship is the key to our success …” was coded as “The importance of safeness.” The first author presented transcripts of meaningful units and discussed preliminary themes with the competence group for feedback. The preliminary themes and associated codes were discussed with the other authors and rearranged into main themes. These themes were discussed with the competence group and the co-researcher and adjusted. The meaningful unit above together with other similar meaningful units was arranged into the subtheme: “To create a safe haven by adapting to the individual needs of each student.” This subtheme was then arranged in the main theme: “To be recognized as a person with strengths and challenges in everyday life.”

**Table I:** Analyzing process.

Meaningful unit	Code	Subtheme	Main theme
“To create a safeness … safeness through a relationship is the key to our success …”	“The importance of safeness”	“To create a safe haven by adapting to the individual needs of each students”	“To be recognized as a person with strengths and challenges in everyday life”
“We work on the engine together, and talk all the time. We talk about everything. We have something in common … In this way we develop relationships.”	“Development of TSR through collaboration and common interests”	“The joys of collaboration in developing common interests”	“Collaborative relationships between teachers and students”

### Ethical approval

The study was approved by the Norwegian Social Science Data Services (NSD). After a complete description of the study to the participants, written informed consent was obtained. All data were made anonymous by moderating or removing details like names and places that could entail the risk of participants being identified.

## Findings

Four main themes were identified in the analysis: (1) to be recognized as a person with strengths and challenges in everyday life, (2) collaborative relationships between students and teachers, (3) flexible boundaries in the relationship between teachers and students and (4) organization of classes and procedures set the stage for TSR (Table II).

**Table II:** Themes.

To be recognized as a person with strengths and challenges in everyday life	Collaborative relationships between teachers and students	Flexible boundaries in the relationships between teachers and students	Organization of classes and procedures set the stage for the TSR
The recognition lies in the small things	Mutuality and responsibility in the relationships	From formal relationships to personal matters	Class size and class frequency influence TSR
To create a safe haven by adapting to the individual needs of each student	The joys of collaboration in developing common interests	Exceeding the expectations	Procedures and meetings that matters
Communicating negative expectations and unresponsiveness		Protecting the boundaries	

### To be recognized as a person with strengths and challenges in everyday life

All focus groups highlighted the importance of teachers responding to the students’ individual needs and to recognize the students in their everyday life. Negative expectations and unresponsiveness from teachers were described as destructive ways of not recognizing the students and responding to their needs.

#### The recognition lies in the small things

Both teachers and helpers described that they thought it was important to show recognition for students in what they described as “the small things.” Some of the teachers said that they call out the name of every student in each class and look them in the eyes to get an impression of how the students are doing each day. Some described how they shake hands with all students every day. The groups of helpers said that small things like looking at the student or asking them questions could be important to make the students feel recognized. One helper said: “It is as simple as saying: Are you doing OK today?” A teacher that had worked for decades said that he decided to do something about his relationship with the students a few years ago. He started to give the students small well-deserved compliments in each class. He found out that it worked very well in developing positive relationships with the students and that he now felt much more satisfied with his work.

#### To create a safe haven by adapting to the individual needs of each student

Being recognized was also described as creating a safeness by acknowledging the individual needs of the students. Safeness was described as the basis of a healthy learning environment but also as a foundation of trust and positive relationships between the teacher and student. One teacher said: “To create a safeness … safeness through a relationship is the key to our success.” Some of the teachers said that safeness makes it easier to talk about difficult things with the students. The helpers described safeness as being especially important to students who have mental health problems. They talked about how the teacher, for example, can avoid asking questions in plenum to students that have problems with anxiety. Both teachers and helpers discussed the special needs of students who had a difficult time at home, had mental health problems and were at risk of dropping out. They described how they had experienced teachers who had been of great support to these students by facilitating and making some special arrangements that made it possible for the students to continue schooling. Some of the helpers described these students’ relationships with their teachers as a rescue. Both helpers and teachers said they had experienced students who looked upon school as a safe haven in a challenging life. One helper said:

I have met students that have had mental health problems and inhuman conditions at home … And the students of course struggle at school … but because they have developed a good relationship with teachers; when the student is laying over the desk and is really tired … so rather than passing negative comments the teacher gives them a supporting hand on the shoulder … The teacher knows what's going on and says: ‘keep up the good work, we will help you’.

The teachers discussed how they could help students that they considered in need of special attention in everyday life. One teacher said:

I think it is important to see the signals in class, and adjust. If you notice that a student is feeling well or not … then you have to consider how much you are going to push them. And maybe you should talk to the student or at least be considerate.

The teachers said that they liked to help each student but some of them found it challenging when they were responsible for a large number of students. The helpers talked about experiences with teachers that do a lot to help students at risk with individual arrangements; they thought this helped these students to stay in school. The teachers also discussed whether there was too much focus on students and their special needs. Some of the teachers with the larger classes had experienced that students sometimes preferred to be rather anonymous in class.

#### Communicating negative expectations and unresponsiveness

The helpers discussed experiences with teachers who had negative expectations for the students. They discussed how this can influence students both on individual levels and as a group. One helper told about an experience he had when he visited a class:

I really reacted to it when I was there. Because she said it all the time: ‘It's just a mess with them, everything is just trouble with them … They just can't get anything right’ … And I thought: If you hear this long enough, then you will become a loser … This is not what they need to accomplish something in life.

They also described teachers that seemed to have negative expectations and sarcastic comments to students that rarely showed up for class. They had experienced that such comments could be hurtful for these students, their relationships with teachers were already poor and it made it even more difficult to show up for school.

The helpers also discussed situations where teachers did not respond to students’ needs. They talked about students that had told them that the teacher did not seem to care when students left in the middle of a class. One informant told about a girl who was bullied in class, they talked to the teacher three times about it before he did anything about the situation. This was very difficult for the student who had a very hard time and was considering quitting school.

### Collaborative relationships between teachers and students

The TSR was described as a mutual relationship between students and teachers; challenges related to the asymmetry of the TSR were discussed. Collaboration in working on common interests and activities was described as a particularly powerful way of developing TSR.

#### Mutuality and responsibility in the relationships

The teachers talked about TSR as a mutual relationship and discussed different challenges related to this. They underlined that students also have a responsibility for a positive TSR. They discussed how they sometimes experienced that students had decided “not to like” the teacher and how challenging this can be. However, they emphasized that there is a strong asymmetry in the relationship and that the teacher always has the responsibility of showing the students’ respect. One of the teachers summed up:

As teachers, we cannot decide to dislike a certain student. That would be completely wrong, because then we would not be doing our job. Therefore, we must … make an effort … but it is challenging. We cannot be best friends with all of them …

However, sometimes teachers meet students that they do not like and whom they find difficult to relate to. Discussing this challenge, the teachers expressed acceptance for the fact that they do not like all students but they feel responsible to treat all students with respect.

The groups of helpers talked about how mutual respect between teachers and students is crucial in building relationships. They addressed the asymmetry of TSR and pointed out the teachers as mainly responsible for the relationships and to make clear what the teachers expect of the students as a basis for mutual relationships.

#### The joys of collaboration in developing common interests

The teachers described how they develop relationships with students as they collaborate in doing a practical task or going hiking together. Teachers from the sports program said that they experienced special and closer relationships with the students when they go on hiking trips and sports events. One of these teachers said:

We sleep in the same tent. We live together, we cook together, and sometimes we go away for one week. It is almost something private. And we as the teachers are allowed to take part. I find it is very important.

Some teachers said that it is easier for them to connect with the students when they have a common interest, for example, sports. These teachers said that they felt that the class environment and relationship with the students in the sports program were especially positive. Teachers in VP had similar experiences. They talked about how they connected and found it easy to learn to know students when they spend several hours a week collaborating: “We work on the engine together, and talk all the time. We talk about everything. We have something in common …. In this way we develop relationships.” Both students’ and teachers’ engagement with the school subjects were described as important in the development of TSR. The teachers said that they establish positive relationships through engagement with the teaching. They highlighted the fact that they are first and foremost teachers and their task is to teach. One teacher stated: “a big part of the relationships is established through the teaching … we should not separate the two ….” Some teachers said that they think that it is difficult to form a good relationship with students that do not like their school subject. Others said that it is possible to develop the relationship through their own engagement with the school subject. The helpers discussed engagement for the school subjects both as a positive factor that motivates students to attend classes and as a negative factor that makes students drop classes when teachers are regarded as bad teachers. One of the helpers said: “When the teacher is good, the student is happy with the school subject. It means a lot. That engagement is also a sign of teachers’ care.” During the discussion of this topic, the helpers talked about the importance of good teachers that promote students’ engagement. Some said that when students are engaged, they will experience that they master schoolwork and it will make them feel better. On the other hand, the helpers had noticed students skipping classes because the students regarded their teacher as uncommitted and bad at teaching.

### Flexible boundaries in the relationships between teachers and students

Both teachers and helpers discussed how they had experienced that the boundaries of TSR had changed over time. They described how a relationship that used to be more distant and professional had developed into a closer and more personal relationship. They discussed possibilities and pitfalls related to closeness and distance in TSR.

#### From formal relationships to personal matters

Both teachers and helpers said that nowadays it is common for students to talk to their teachers about difficult and personal challenges in their lives. One of the teachers who had worked in the field for decades said: “When I started out as a teacher the students called me by my surname, nowadays they tell me about trouble in their love life and expect me to make special arrangements when they have a heart-ache.” They discussed experiences of a development over the past decade to more openness regarding students’ emotional matters and mental health. One teacher said: “In the old days the focus was solely on the academic part, but now the focus is more on the student as a person.” The participants reflected upon whether this development could be a reflection of a development in society in general. Both teachers and helpers said that they now experience more equal and open relationships between teachers and students.

#### Exceeding the expectations

The participants brought up experiences related to TSR where teachers did something extraordinary for some students. One teacher said that she had promised a student that had dropped out several times before, a small present if he stayed in school this year. This was partly a joke in class but she now, halfway through the year, experienced that the student still showed up for school. Another teacher told about how he went with a student to a police interrogation because he knew that the student had no one else to go with him. The helpers told stories of teachers that would regularly pick up students every morning on their way to school because they were worried that students would dropout. They also described how teachers helped students with small things like postponing a test or handing over some papers the students forgot. They reflected upon how this could be of great help to students that struggle and are at risk of dropping out.

#### Protecting the boundaries

Teachers also brought up challenges related to closeness and how it was important to protect the boundaries between teachers and students. They described how it could be difficult for the teachers to draw a line when students have personal problems and seek a lot of contact. Several of the teachers stated that they are not psychologists; they are not supposed to offer treatment and sometimes they found it hard to know how to handle the students’ problems. One teacher said:

I have had students calling me in the evening. They have had mental health problems. Sometimes it is a dilemma, because they do not have many others to turn to. But I don't know what's best for them when they are ill …

Both the teachers and the helpers described that some teachers worry a lot about their students. Some of the helpers said that the teachers are some of the first to know if something is wrong with the students because they know them so well and see the students frequently. The primary contact teacher will often have a meeting with the students if they worry about them, but both teachers and helpers said that it is important to have a distinction between talking to the students about their problems and treating mental illnesses. All groups highlighted that experts should treat mental health illness. One helper said: “When it comes to someone who needs treatment … then a different professional has to take over. Psychologist and school nurses are the ones who know this profession ….” Some teachers said that they would contact the school nurse when they were worried about the students’ mental health while others found it difficult to know when it is necessary to get assistance.

Both teachers and helpers emphasized the importance of not getting too close with all students. Some said that some students do not want close relationships with their teachers.

One experienced teacher said:

I was too close, and that relationship has been a disaster for over a year now. But it looks like it's getting a little bit better now. He didn't want to talk to me for over a year …

Another teacher said: “And some of them I don't want to push myself onto sometimes, because some create a brick wall around themselves, others are more inviting. And that's the way it is.” The teachers highlighted the importance of respecting students’ wishes of wanting to keep things private.

### Organization of classes and procedures set the stage for the TSR

All groups discussed structural conditions such as organization of classes and different school procedures and meeting as important factors contributing to the forming and development of TSR.

#### Class size and class frequency influence TSR

The difference in organization of VP and SGS was described as particularly important for the development of TSR. In SGS, the maximum number of students in each class is 30, whereas in vocational classes the maximum is 15. The organization of the different programs and classes lead to big differences in how many students each teacher teaches. Some teachers in gym and geography said that they have the students for 2 h a week; others typically in the VP have the same students for 10–15 classes a week. Therefore, there were big differences in how many students the teachers had to relate to. Some of the vocational teachers said that they had 12 students in total this year, while the gym teachers said they had up to 200 students. Both teachers and helpers discussed how this organization influenced the TSR. One teacher from a VP said: “Because you have so many classes with them. So this relationship … I don't want to say it's better, but you at least have the possibility to create that safeness, trust and to acknowledge each student ….” Other teachers talked of how it was possible to connect with 30 students when they have many classes with the same students: “I think I have time to talk to all 30 students in class …. I walk around the classroom and sit down beside them … we have 10 classes a week together ….” Both teachers and helpers discussed how the organization of VP gave the teachers the opportunity to follow the students more closely. As one helper put it: “The most important thing is that students feel that the teachers recognize them, both regarding the school subjects and especially psychosocially. That seems easier when there are fewer students in class.”

#### Procedures and meetings that matters

The influence of school organization and procedures on TSR was discussed in all groups. All upper secondary schools have a system where each student has a primary contact teacher who has a special responsibility to follow-up with the individual student. The primary contact teachers are obliged to have structured meetings following certain procedures with their students every year. Some of the helpers emphasized the importance of the structure of these meetings. One helper said:

They are obliged to have these meetings with the students … The primary contact teacher should allocate ample time for the meeting and conduct a good meeting … The management at school tries to quality assure these meetings, so this is not entrusted to coincidences and to the individual teachers.

On the other hand, critical views about these procedures and the importance of the informal conversations with the students were also presented. Like this teacher:

… that first meeting, I don't think we get a lot of personal insight. It is a bit superficial, I think. When we are supposed to follow these forms and tick in some boxes. It is more in the informal conversations at recess, when you meet someone in the corridor, at the library and grab them … or they grab the teachers … There is much more to these conversations …

A helper said:

I am getting worried about the abstraction of useful approaches … It's like once you have a plan and put it on a paper then you are saved. We know that engagement is what helps, personal engagement, to show an actual interest for the person you are confronting ….

The teachers also brought up how they felt obliged to prevent students from dropping out. They explained how dropout prevention was highly emphasized by the management as a determination of success and that the schools’ budgets depend upon students’ graduation.

—and we are obliged to get them through … in a way … and we cannot just let them go because the statistics are something that are used to determine whether a school is successful … — and the economy … because of the budget, it comes with the student.

Social workers, school nurses and school advisors were described as important collaborators and as resources in helping students with both mental health problems and risk of dropping out. Network meetings were frequently used as a collaborative intervention for these students. These meetings are typically held once a month and include the student, the primary contact teacher, parents, school advisor and school nurse. The meetings were described as a contribution to promote tighter and more positive relationships between students and teachers. One helper reflected upon this: “The students’ have positive experiences with these meetings because they notice that they get a completely different approach and a close relationship with their primary contact teacher. And they will get much more help in everyday life.”

## Discussion

In this study, we have explored how teachers and helpers experience that TSRs are developed and promoted in upper secondary school. We have also explored their experiences of important relational qualities concerning students’ mental health and dropout in upper secondary school.

The participants in this study described how they found it important for teachers to recognize each student and facilitate safeness in students’ everyday life. Communicating negative expectations and unresponsiveness were described as negative qualities of TSR. Collaboration between teachers and students in working together on common interests and practical tasks was described as an important quality of developing TSR. The participants reflected upon the change in expectations from students regarding the boundaries of TSR, and it had developed over time and now involves more personal matters and closer relationships. Situations where teachers do something extraordinary for students were described as positive, but challenges related to closeness in the relationship were also brought up. Contextual matters like class size, frequency and collaborative meetings were described as setting the stage for TSR. We present our discussion around the emotional and collaborative qualities of TSR. We will also discuss some of the contextual factors contributing to the development of TSR.

In our analysis, we have identified several emotional qualities related to TSR: safeness, recognition, closeness, unresponsiveness and negative expectations. To facilitate safeness was emphasized as especially important to students with mental health problems. Considering the fact that youth with mental problems often have problems with anxiety and insecurity, a TSR that facilitates safeness seems important. Other studies have identified safeness as an important component of students’ school climate that influences students’ life satisfaction in a positive way (Suldo, Riley, & Shaffer, 2006). Creating a safeness can potentially also prevent dropout as other studies have shown that feeling unsafe is a risk factor for students staying at home and eventually dropping out of school (Gietz & McIntosh, 2014). Based on these findings, it appears to be an interplay between feeling safe, life satisfaction in general, mental health and dropout.

Recognition and responding to students’ personal needs were highlighted as important in our study. Adolescence is a vulnerable time in life and recognition by other adults can be crucial in protecting young people at risk. Students that have dropped out of school have reported that they felt invisible and overlooked by teachers and the school system (Natland & Rasmussen, 2012). Students with mental health problems often refrain from asking for help at school because of low self-esteem, anxiety or negative thinking patterns. By recognizing and adapting to their needs, these students might get the help they need to stay in school. Our study showed that recognition often lies in the small details of everyday interactions like asking the students if they are ok. This finding is in line with studies from the mental health field that emphasize the importance of “the small things” that make a big difference (Davidson & Johnson, 2013; Ness, 2016). Other studies have found emotional support, care, empathy and warmth as important concepts to promote students’ mental health and prevent dropout (Cornelius-White, 2007; Croninger & Lee, 2001; Langaard & Toverud, 2009; Wang et al., 2013). Although these emotional concepts were not identical with the concept of recognition and adapting to students’ needs, they were similar and reflect the complexity of emotional qualities of TSR.

Communicating negative expectations and unresponsiveness were found to be negative qualities of TSR in our study. This is in line with other studies that have identified the negative concepts of “labeling” and “judgment” (McGrath, 2009; Muller, 2001). These concepts are referring to situations where the self-identity and behavior of the students are influenced by the terms used to describe or classify them in negative ways. “Labeling” and “judgment” were related to negative TSR and have the potential to lead to lower self-esteem for students, disconnection with school and eventually dropout (McGrath, 2009; Muller, 2001). This was supported by our study where the participants described students that did not show up for school because of negative relationships with teachers. These findings are especially worrisome as negative TSR has been found to be quite stable and persistent over time (Roorda et al., 2011). In addition, students at risk often have more negative TSR than normative students (Drugli, Klökner, & Larsson, 2011; Hamre & Pianta, 2001).

Our analysis showed that collaboration is regarded as important in developing TSR. In our findings, teachers especially in VP highlighted the importance of developing relationships through collaboration on practical tasks and hiking trips. The collaboration of working on common interests and engagement for the school subjects were described as something that promoted positive relationships, common experiences and informal talks. Studies of resilience in youth emphasize the power of the ordinary processes in the everyday life of youth and children (Masten, 2001). In our findings, the informal talks and collaborative ways of spending time together represent ordinary processes in youths’ everyday life that have the potential to influence both students’ mental health and dropout. Collaboration is essential in establishing helping relationships. Other studies have shown that collaboration and informal talks are crucial in recovering from mental illnesses (Borg & Topor, 2014; Karlsson & Borg, 2013). Collaboration and informal talks between teachers and students may have some of the same potential. A study by Croninger and Lee (2001) found that informal talks between teachers and students were strongly related to reduce dropout in students at risk. Interventions of prevention of dropout and promotion of students’ mental health often emphasize special interventions and treatment, the ordinary processes like collaboration and small-talk are often underrated.

Our findings showed that the teachers experience a prevailing expectation from students to be involved on a closer and more personal level with their students. Other studies have also found that closeness in TSR is particularly important to students as they grow older (Roorda et al., 2011). At the same time, there is an increasing focus in society on academic achievement and reduction in dropout rates (Markussen, Froseth, & Sandberg, 2011; Mausethagen, 2015). An academic focus and a relational focus are often presented as dichotomous. However, many studies indicated that teaching based on both relational and academic support produce the best academic results (Hattie, 2009; Nordenbo et al., 2008). In our study, the joys of collaborating together on common interests showed how teachers and students develop relationships through teaching and how these concepts are intertwined. The findings in our study highlighted the important role teachers have of promoting mental health in students’ everyday life by recognizing the students as persons. Nevertheless, our findings also showed that it can be demanding for teachers to balance the closeness and personal dimensions regarding students’ mental health issues. This is in line with other studies that have found that teachers are struggling to deal with the complexity of their roles and the many expectations for them (Graham, Phelps, Maddison, & Fitzgerald, 2011; Mausethagen, 2015).

Our findings suggested that contextual factors like class size and frequency of classes contribute in setting the stage for TSR. These findings underline the importance of a developmental systems understanding of the TSR where different multilevel factors interact in the development of TSR (Bronfenbrenner & Morris, 1998). Class size has been frequently studied and discussed. Hattie (2009) concluded in his meta-study that class size was not significant for students’ performance and achievement. On the other hand, the same meta-study showed that TSR and the time teachers spent with students were significant (Hattie, 2009). However, in our study we are looking at the TSR in particular relation to students’ mental health and dropout. The structural factors like class size and frequency affect how many students each teachers relate with. As our findings showed that the participants express an increasing expectation for more personal TSR, recognition of each student and adapting to students’ individual needs it sounds demanding to achieve this for teachers that relate to 200 students. It seems likely that teachers that relate to 12 students have better opportunities to recognize each student and adapt to their individual needs. Likewise, it is easier to get to know the students when the classes are more frequent.

### Limitations and reflexivity

The first author who carried out the interviews in this study has worked as a clinician within youth mental health care. This brings the possibility of a bias in understanding the participants’ experiences in light of former experiences. On the other hand, the researcher's experiences can also make it easier to familiarize with the scope of the study.

The study used a participatory approach. However, there was an imbalance between the professional researchers and the co-researchers in the degree of involvement, educational background and perspectives that affect their influence in the study may hinder true participative involvement in the research process. Bringing in a young co-researcher with lived experiences from dropout is supposed to contribute to reflexivity in the study by, for example, contributing in reframing the questions in the interview guide. However, some of the participants may not feel free to express their experiences with TSR when a young co-researcher is present during the interviews.

Traditionally, participatory research has been viewed as biased; however, one could also argue that all researchers have an impact on the research process (Veseth, Binder, Borg, & Davidson, 2012). In this study, we aimed to use the researchers’ subjectivity as an opportunity to understand the participants in a broader perspective. As this approach demands reflexivity both researchers and co-researchers focused on being aware of our preconceptions in this study, we reflected together on how this influenced the research process.

A limitation in using focus groups as a method of exploring the TSR lies in the problem of distinguishing between the participants’ expressed experiences of TSR and their ideas of what would be an ideal TSR.

## Conclusion

In this study, we have found that teachers and helpers describe and experience collaborative, emotional and contextual qualities as important in the development of TSR in upper secondary school. The findings of our study suggest that teachers and helpers experienced that TSR may have the potential to play a role in promoting mental health in students’ everyday life. The findings showed how teachers can support students with mental health problems. However, the findings also indicated that the participants have experienced that negative qualities of TSR can contribute to push students out of school. On the other hand, students can find school to be a safe haven when the TSR is safe and adapted to their personal needs.

The interplay between contextual factors and the development of TSR calls for awareness from decision-makers and school management to facilitate structures that promote positive TSR. A challenge here seems to be how schools can provide more supportive cultures for the teachers who struggle to balance their roles as both educators and supportive adults. The findings of positive and negative emotional qualities of TSR call for awareness in teachers’ educational programs. Teachers should focus on developing strategies on how to avoid negative emotional qualities of TSR like unresponsiveness, but also how to develop positive qualities of TSR like safeness. The findings of this study call for a higher awareness of the informal everyday processes, the collaboration between students and teachers and how this may influence students’ lives.
